# MVPANI: A Toolkit With Friendly Graphical User Interface for Multivariate Pattern Analysis of Neuroimaging Data

**DOI:** 10.3389/fnins.2020.00545

**Published:** 2020-07-08

**Authors:** Yanmin Peng, Xi Zhang, Yifan Li, Qian Su, Sijia Wang, Feng Liu, Chunshui Yu, Meng Liang

**Affiliations:** ^1^School of Medical Imaging, Tianjin Medical University, Tianjin, China; ^2^Tianjin Key Laboratory of Functional Imaging, Tianjin Medical University, Tianjin, China; ^3^Department of Radiology, Tianjin Medical University General Hospital, Tianjin, China

**Keywords:** machine learning, multivariate pattern analysis, multivoxel pattern analysis, neuroimaging, graphical user interface, data fusion, classification, regression

## Abstract

With the rapid development of machine learning techniques, multivariate pattern analysis (MVPA) is becoming increasingly popular in the field of neuroimaging data analysis. Several software packages have been developed to facilitate its application in neuroimaging studies. As most of these software packages are based on command lines, researchers are required to learn how to program, which has greatly limited the use of MVPA for researchers without programming skills. Moreover, lacking a graphical user interface (GUI) also hinders the standardization of the application of MVPA in neuroimaging studies and, consequently, the replication of previous studies or comparisons of results between different studies. Therefore, we developed a GUI-based toolkit for MVPA of neuroimaging data: MVPANI (MVPA for Neuroimaging). Compared with other existing software packages, MVPANI has several advantages. First, MVPANI has a GUI and is, thus, more friendly for non-programmers. Second, MVPANI offers a variety of machine learning algorithms with the flexibility of parameter modification so that researchers can test different algorithms and tune parameters to identify the most suitable algorithms and parameters for their own data. Third, MVPANI also offers the function of data fusion at two levels (feature level or decision level) to utilize complementary information contained in different measures obtained from multimodal neuroimaging techniques. In this paper, we introduce this toolkit and provide four examples to demonstrate its usage, including (1) classification between patients and controls, (2) identification of brain areas containing discriminating information, (3) prediction of clinical scores, and (4) multimodal data fusion.

## Introduction

Multivariate pattern analysis (MVPA), a machine learning technique used in neuroimaging data analysis, has rapidly grown in popularity in recent years (Liang et al., 1993; Dosenbach et al., 2010; Wager et al., 2013; Liu et al., 2015, 2017; Moradi et al., 2015; Meng et al., 2016; Hazlett et al., 2017; Chung et al., 2018; Camacho et al., 2019). Compared with traditional univariate analyses, such as the general linear model (GLM), MVPA has been shown to be more powerful in information detection as well as in clinical applications for several reasons. First, MVPA examines the spatial pattern of signals sampled from multiple voxels rather than the signal of a single voxel at a time; thus differences in signal spatial distribution within a brain area between two experimental conditions can be detected even when the overall average signal amplitude of this area does not differ between the two conditions. Second, MVPA naturally avoids or reduces the statistical problem of multiple comparisons (Durnez et al., 2014; Chen et al., 2018) by examining multiple voxels at once and, thus, is statistically more powerful. Third, MVPA can be used directly for diagnosis or prognosis for a new patient based on pre-trained models and is, thus, more relevant for clinical applications, such as personalized medicine. Some detailed explanations for why MVPA is more powerful can be found in several review papers (Mur et al., 2009; Mahmoudi et al., 2012; Haxby et al., 2014; Haynes, 2015; Kragel et al., 2018).

To facilitate the application of MVPA technique in neuroimaging studies, several specialized open-source software packages have been developed based on some popular programming platforms, such as MATLAB, Python, or Java (LaConte et al., 2005; Hanke et al., 2009; Pyka et al., 2012; Schrouff et al., 2013; Grotegerd et al., 2014; Hebart et al., 2014; Oosterhof et al., 2016; Bode et al., 2019). Although these existing software packages are excellent and have made significant contributions to facilitating the application of MVPA technique in the neuroimaging field, most of them are based on command lines rather than graphical user interfaces (GUIs). Lacking a user-friendly GUI obviously limits the use of MVPA technique with neuroscientists or clinicians who do not have experience in programming. For those who only have basic programming skills, although simple MVPA analyses may be manageable, it is still difficult to perform relatively more complex analyses and makes full use of this technique. Moreover, without a standard user interface, every study designs its own parameters and programming scripts, which are often unavailable to others, and thus, it is relatively difficult to compare or replicate results from different studies without programming another script that should be identical to the script used in other studies. Therefore, an MVPA software toolkit specialized for neuroimaging data analysis with a user-friendly GUI is in urgent need in the field. To our knowledge, the existing free software toolkits (MANAS, Rana et al., 2013; PRoNTo, Schrouff et al., 2013; and MANIA, Grotegerd et al., 2014) have developed GUIs. However, choices of machine learning algorithms or formats of input data files are limited in these toolkits. For example, only NIFTI (e.g., files with the extension “.nii”) and ANALYZE (e.g., files with the extension “.hdr/img”) file formats are allowed in these software packages (MANIA additionally allows SPM.mat from the SPM software as input files), and thus, brain connectivity, behavioral, or clinical measures cannot be easily analyzed using these software packages.

Another growing interest in the neuroimaging field is to combine MVPA with data fusion, such as fusing different types of imaging measures (e.g., fusing structural and functional measures, or fusing activation and connectivity measures) or fusing imaging with non-imaging measures (e.g., genetic, behavioral, or clinical measures). Indeed, it has been shown that complementary information contained in different types of data can be fused to improve classification or predication performance (Walhovd et al., 2010; Zhang and Shen, 2012; Liu et al., 2014; Cabral et al., 2016; Qureshi et al., 2017; Tong et al., 2017; Tulder and Bruijne, 2018). Currently, only PRoNTo offers the function of data fusion using multiple kernel learning, and no other data fusion methods are available (Schrouff et al., 2018).

Therefore, we developed a new GUI-based software toolkit (MVPANI) for neuroimaging data analysis using MVPA with more options of machine learning algorithms and data formats as well as the function of data fusion. As with many other neuroimaging data analysis toolkits, such as statistical parametric mapping (SPM^1^) and DPABI (Yan et al., 2016), MVPANI is developed based on the MATLAB platform, which is familiar to most neuroimaging researchers. It also utilizes functions of other existing software packages such as libsvm (Chang and Lin, 2011), the decoding toolbox (Hebart et al., 2014), and SPM. MVPANI includes a comprehensive range of functionalities, such as feature preprocessing, data fusion, classification, and regression with a range of machine-learning algorithms and statistical testing and can output a range of results files. A detailed comparison between the existing software packages and the MVPANI presented in this paper is provided in Table 1. In this paper, we introduce this software package and also provide four examples to demonstrate the usage of MVPANI in different situations, including (1) classification between patients and healthy controls, (2) identification of brain areas containing discriminating information, (3) prediction of clinical scores of patients, and (4) multimodal data fusion.

**TABLE 1:** Updated comparison of existing free software packages for multivariate pattern analysis of neuroimaging data, based on Schrouff et al. (2013) and Grotegerd et al. (2014).

**Software**	**Author**	**Inputs**	**Multiclass**	**Classifiers**	**Regression**	**Feature selection**	**Data fusion**	**Primary language**	**Interfaces**	**GUI for results**
3dsvm	LaConte et al. (2005)	AFNI images: fMRI data	Yes	SVM	No	Masking	No	C	Basic GUI/Command line	No
PyMVPA	Hanke et al. (2009)	NumPy arrays, text, NIfTI images, EEP binary file	Yes	KNN, SVM, SMLR, GP, PLR, GLM, RR, RF, ExtraTrees, Searchlight	GP, LARS, PLR, RR, SMLR, ExtraTrees, GLM, RF	Masking, Filters, Wrappers; PCA	No	Python	Command line	No
Weka Interface	Pyka et al. (2012)	NIfTI images	Yes	SVM, GP, NB, DT, KNN	SVM, GP	Filters, Wrappers	No	Java	GUI	Yes
PRoNTO	Schrouff et al. (2013)	NIfTI images	Yes	Binary SVM, binary and multiclass GP	GP, RVR, KRR	Masking	L1-Multiple Kernel Learning	Matlab	GUI/Command line	Yes
MANAS	Rana et al. (2013)	NIfTI images	No	SVM, Searchlight	No	Masking	No	Matlab	GUI	Yes
MANIA	Grotegerd et al. (2014)	NIfTI images, SPM.mat file	Yes	SVM, GP, KNN, LDA, NB, ensemble methods	No	Masking, Filters, Wrappers, Embedded	No	Matlab	GUI	Yes
The Decoding Toolbox	Hebart et al. (2014)	NIfTI images, mat file	Yes	SVM, LR, correlation classifier	SVM, LDA	Masking, Filters, Embedded; PCA	No	Matlab	Command line	No
CoSMoMVPA	Oosterhof et al. (2016)	NIfTI or GIFTI images, M/EEG	Yes	LDA, SVM, KNN, GNB, Searchlight	No	Masking	No	Matlab/GNU Octave	Command line	No
MVPANI	In this paper	NIfTI or GIFTI images, mat or txt files	No	SVM, KNN, LR, LDA, NB, DT, RF, Searchlight	SVM, KNN, LR, NB, DT, RF	Masking, Filters, Wrappers, Embedded; PCA	Yes	Matlab	GUI	Yes

*LDA, linear discriminant analysis; LR, logistic regression; KNN, K-nearest neighbors; (G)NB, (Gaussian) naive Bayes; DT, decision tree; RF, random forest; GUI, graphical user interface; GLM, general linear model; SVM, support vector machine; GP, Gaussian processes; LARS, least angle regression; (K)RR, (Kernel) ridge regression; RVR, relevance vector regression; PLR, penalized logistic regression; SMLR, sparse multinomial logistic regression; LASSO, least absolute shrinkage and selection operator; RFE, recursive feature selection. Note that Weka Interface (http://www.unimarburg.de/fb12/kebi/research/software/nifti_importer) is not available for download any more.*

## Software Structure of Mvpani

**FIGURE 1:**
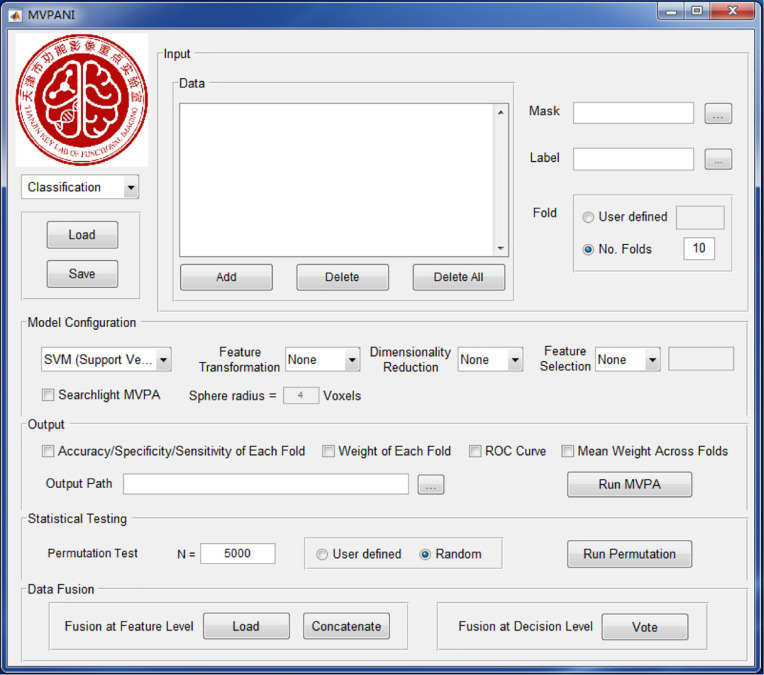
The GUI of MVPANI. It consists of five main modules: input, model configuration, output, statistical testing, and data fusion.

**FIGURE 2:**
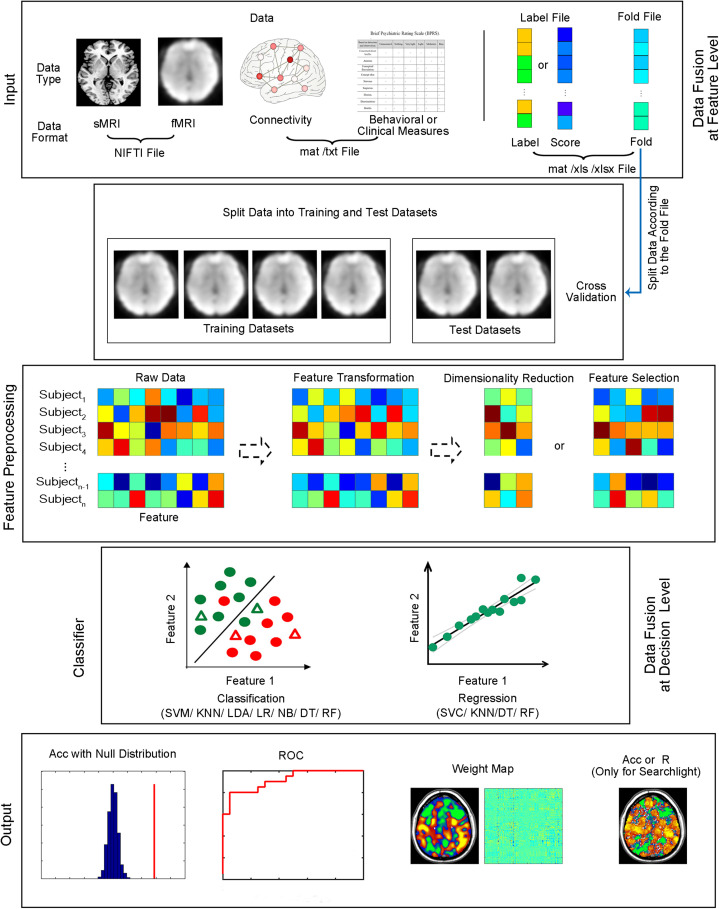
Structure of MVPANI. Different types of data with different file formats are allowed in MVPANI. The categories or continuous scores of all samples can be specified in the label file. The samples are further divided into a training dataset and test dataset according to the fold file. The raw data can be preprocessed, such as feature transformation, dimension reduction, and feature selection, before being fed into classification or regression models. Multiple machine learning algorithms are available for performing classification or regression tasks. Results can be obtained in a variety of formats, including classification accuracies (or prediction precisions) along with their statistical significance, the ROC curve, and weight maps. In addition, searchlight MVPA is also implemented in MVPANI. Particularly, different types of data can be combined to improve MVPA performance using two data fusion strategies; data can be fused at the feature level or at the decision level. Acc, classification accuracy. R, correlation coefficient between the predicted values and the actual values, as a measure of prediction precision, when performing regression tasks.

The current version of MVPANI requires MATLAB R2015b. The software can be launched by entering “MVPANI” in the command window of MATLAB after adding its path. Both classification and regression are implemented in MVPANI and use a common main GUI, which is composed of five modules: data input, model configuration, statistical testing, results output, and data fusion. The main GUI and an overview of the basic structure of the toolbox are shown in Figures 1, 2, respectively. We describe the structure and the key functions of every module in the following sections.

### “Input” Module

This module specifies the data files of all samples and their label information that are used in the subsequent MVPA. MVPANI allows inputs of several different file formats, including NIFTI files, GIFTI files (i.e., with the extension “.gii”), ANALYZE files, Text files (i.e., with the extension “.txt”), and MATLAB data files (i.e., with the extension “.mat”). These formats can accommodate all neuroimaging-related data—all image-type data can be represented using .nii, .gii, or .hdr/img file formats, such as structural or functional brain maps, and all non-image-type data can be represented using a .txt or .mat file format, including connectivity matrices, network topological measures, genetic data, or even behavioral or clinical measures. If .txt or .mat files are used, the data should be stored as a two-dimensional matrix of which rows indicate samples and columns indicate features. If only a subset of features are to be used in the subsequent MVPA, a binary mask file in any of the above formats can be specified to indicate the features of interest.

The label information of all samples can be arranged as a column vector (each row represents a sample) and stored in a .txt, .mat, or .xls/xlsx file. For classification, the label indicates the true class of each sample. If you have two classes, the labels can be set as 1 or –1 (or 1 or 0). For regression, the label file contains continuous values corresponding to the actual score obtained from each sample.

As MVPA typically adopts a training–testing procedure, the training and test datasets need to be defined before running machine learning algorithms. For example, to run a ten-fold leave-one-out cross-validation, all samples need to be divided into 10 folds (i.e., 10 groups); every fold is used as a test dataset once (that is, in each cross-validation step, one fold is used as the test dataset, and the remaining nine folds are used as the training dataset). There are two ways to specify the “fold” information in MVPANI: (1) Users can simply enter the number of folds, and the software automatically and evenly divides all samples into different folds in a random manner; (2) alternatively, a user-defined “fold” file (of a format of .txt, .mat, or .xls/xlsx) can also be entered. Similar to the label file, the fold file also contains a column vector of which each row represents to which fold the corresponding sample belongs.

### “Model Configuration” Module

This module includes specification of machine learning algorithms and feature preprocessing methods.

#### Machine Learning Algorithms

Seven algorithms have been implemented in the current version of MVPANI; all can be used for solving classification problems, and four of them can be used for solving regression problems.

##### Support Vector Machine (SVM)

Support vector machine (SVM) (Cortes and Vapnik, 1995) is one of the most commonly used machine learning algorithms in neuroimaging data analysis. SVM has been proven to work well in situations in which the number of samples is far less than the number of features and is, thus, very suitable for typical neuroimaging data; there are usually tens or hundreds of subjects (i.e., samples) and tens of thousands of brain voxels or connectivities (i.e., features). SVM can be used for both classification (i.e., support vector classification, SVC) and regression (i.e., support vector regression, SVR). For classification, SVM looks for a hyperplane that separates the two classes of training data with a maximal distance between the hyperplane and the training samples closest to the hyperplane (i.e., the support vectors), represented by the following equations:

$$\omega^{T}\,x+b=0$$

$$\max_{\omega ,\text{b}}\frac{2}{||\omega ||}$$

$$\text{s}.\text{t}.y_{\text{i}}\,\left(\omega^{\text{T}}+\text{b}\right)\geq 1,i=1,2,\cdots ,\text{m}$$

where *x* is the feature vector; ω is the weight vector that determines the direction of the hyperplane; and *b* is the displacement term that determines the distance between the hyperplane and the origin. In this way, the interval between the samples of the two classes is the largest so that the two classes are most separable by this hyperplane. The class membership of a test sample is determined by the hyperplane obtained during training. In the case of linearly inseparable data, SVM maps the input space to a high-dimensional feature space by kernel functions to improve the separability of the data. For regression, it is also to find an optimal regression plane that best fits all training data samples. In MVPANI, SVM is implemented using the libsvm toolbox (Chang and Lin, 2011). With a GUI, different types of SVM along with their key parameters (e.g., C-SVC or v-SVC, e-SVR, v-SVR) and different kernels (e.g., linear vs. several non-linear kernels) can be flexibly specified by users. By default, C-SVC with a linear kernel is used for classification, and e-SVR with a linear kernel is used for regression. Default values are also set for common SVM parameters (penalty coefficient *c* = 1, gamma *g* = 0.1, degree *d* = 3, coefficient *r* = 0, nu *n* = 0.5, the epsilon in loss function *p* = 0.1), which should be suitable for most situations. Alternatively, values of SVM parameters can also be specified by users through GUI.

##### K-Nearest Neighbors (KNN)

K-nearest neighbors (KNN) (Cover and Hart, 1967) is a classic machine learning algorithm and can be used for both classification and regression. For classification, the class membership of a test sample is determined by the class memberships of its *k* nearest neighbors: the given test sample is assigned to the class to which most of its nearest neighbors from the training dataset belong. For regression, the predicted value of a test sample is determined by averaging the values of its *k* nearest neighbors from the training dataset. The nearest neighbors are defined by the Euclidian distance between samples in the feature space. The number of nearest neighbors is set to 11 by default or can be defined by users through the GUI in MVPANI.

##### Logistic Regression (LR)

Logistic regression (LR) (Theil, 1992) is also widely used in solving classification problems. This method fits the training data to a sigmoid function, which describes the probability of the occurrence of an event:

$$h_{\omega }\,\left(x\right)=\frac{1}{1+e^{\omega^{T}\,x}}$$

where *x* is the feature vector and ω is the weight vector. A test sample’s class membership is determined by applying a threshold to the estimated probability: the given test sample is classified as category A if the probability is greater than 0.5 or as category B otherwise.

##### Naïve Bayesian Classification (NBC)

Naïve Bayesian classification (NBC) (Domingos and Pazzani, 1997) is a classification method based on Bayes’ theorem. In NBC, the posterior probability function describing how likely a sample belonging to each class is first estimated using the training datasets. The class membership of a test sample is determined by the posterior probability of the test sample belonging to a certain class given the observed data: the test sample is assigned to class A if the posterior probability corresponding to class A is the greatest compared with those corresponding to other classes. As with LR, NBC can also provide a probability measure of a sample belonging to a particular class.

##### Linear Discriminant Analysis (LDA)

Linear discriminant analysis (LDA) (Belhumeur et al., 1997), also known as Fisher’s linear discriminant (FLD), is for solving classification problems. It linearly projects the training data from the feature space to an optimally discriminating space where there are minimal within-class variance and maximal between-class variance so that an optimal classification hyperplane can be defined. The class membership of the test samples can then be determined by this trained hyperplane in the discriminating space.

##### The Decision Tree (DT)

The method of decision tree (Breiman et al., 1984) is for solving both classification and regression problems. A decision tree consists of three main components, namely root nodes, internal nodes, and leaf nodes. The decision-making process starts from the root node and ends at the leaf nodes. The key in constructing a decision tree is to select the best feature as the node to split samples into two groups with the highest purity in each step, which is based on the Gini index for solving classification problems or the mean-square error (MSE) for solving regression problems (Breiman et al., 1984) in MVPANI.

##### Random Forest (RF)

Random forest (Breiman, 2001) can be considered as a collection of multiple decision trees and can be used for solving both classification and regression problems. Each decision tree is created by using only a subset of original samples and features selected by random sampling with replacement. Each decision tree can provide a decision for a test sample. For classification, the final decision is determined as the majority of the decisions of all decision trees. For regression, the final predicted value is determined as the average value of all decision trees. In MVPANI, the number of decision trees is set to 500 by default or can be specified by users.

#### Feature Preprocessing

Before data are entered into a selected machine learning algorithm, the data can be preprocessed as needed. MVPANI provides three types of feature preprocessing: feature transformation, dimension reduction, and feature selection.

##### Feature Transformation

Feature values can be normalized to *Z* score (i.e., zero mean and unit variance) using the following formula:

$$Z=\frac{x-m}{\sigma }$$

where *m* and σ are the mean and the standard deviation, respectively, of the training dataset only, which are used for normalization of both the training and test datasets. This transformation can be performed across rows (i.e., across samples) or across columns (i.e., across features) as specified by users.

##### Dimension Reduction

Neuroimaging data typically have very high dimensions and it is often beneficial to reduce the dimensionality by mapping the original high-dimensional feature space to a low-dimensional space before running MVPA. Indeed, the original high-dimensional feature space usually contains more irrelevant or redundant information, which can be reduced after reducing the dimension and, thus, can potentially improve the MVPA performance. MVPANI currently provides principal component analysis (PCA) for dimension reduction. PCA (Jolliffe, 1986) is a method that decomposes the original data into several principal components (PCs) orthogonal to each other. Each PC is a linear combination of the original dimensions and captures the direction in which the original data has maximal variance. Using PCA, the dimension is reduced by keeping only the PCs with large variances and discarding the directions with small variances. Therefore, PCA reduces the dimension while preserving as much variance of the original data as possible. By default, the number of PCs (*n*) that are kept is determined as such: Top *n* PCs explain ≥95% total variance, and top *n −* 1 PCs explain <95% total variance. Alternatively, the number of PCs that are kept can also be specified arbitrarily by users. Note that, PCA is only performed using the training dataset, and the resultant principal component load coefficient matrix is further applied to the test samples to reduce the dimension of the test dataset.

##### Feature Selection

The purpose of the feature selection procedure is to select only “useful” features and to remove irrelevant or redundant features from the original feature space. Therefore, similar to dimension reduction, feature selection can also reduce data dimension with the effect of removing noise and redundant information and also reducing computational load. However, unlike dimension reduction, in which the resulting features (e.g., the PCs) do not correspond to original features, but a transformation of all original features, the remaining features selected by the feature selection procedure are still original features without any transformation. MVPANI provides two ways to select useful features: (1) least absolute shrinkage and selection operator (LASSO), and (2) a predefined number of selected features based on *F* scores or feature weights.

LASSO performs the L1 regularization to obtain a sparse model with the goal of minimizing the MSE of the predicted values, such as the following objective function:

$$\min_{\omega_{0},\omega }\frac{1}{2\,N}\,\sum_{i=1}^{N}{\left(y_{i}-\omega_{0}-x_{i}^{T}\,\omega \right)}^{2}+\lambda \,\sum_{j=1}^{p}\left|\omega_{j}\right|$$

where the parameter λ is determined by a ten-fold cross-validation within the training dataset resulting in a minimal MSE in MVPANI.

Users can also predefine the number of selected features based on the discriminative power of each feature measured by *F* scores or weights. The *F* score of a feature measures how discriminative between two classes this given feature is: the higher the *F* score, the more discriminative the given feature. It is calculated as the *F* value of an *F* test comparing training samples between two classes. Feature weights are obtained by training a classification or regression model using the training dataset. The weight of a feature reflects the contribution of this given feature to classification or regression: the higher the weight, the more important the given feature to the classification or regression. After ranking all features according to their *F* scores or weights, a predefined number of features with the highest *F* scores or weights can be selected and entered into the subsequent MVPA. The amount of selected features can be specified by users either as an exact number of features to be selected or as a percentage of the total number of features. Note that, feature selection (i.e., the calculation of *F* scores or feature weights) is based only on the training dataset. As it is often difficult to predefine a suitable number of selected features, users are allowed to enter a series of predefined numbers of selected features in MVPANI for convenience. This generates a series of selected feature sets, and multiple independent MVPA are automatically performed for every feature set. However, to avoid “cheating,” users should not simply report the best classification accuracy among those obtained from all selected feature sets; instead, this procedure should be considered as multiple independent MVPA analyses, and thus, all obtained classification accuracies should be reported, and the corresponding statistical significance (i.e., the *P* values) should be corrected for multiple comparisons (e.g., using Bonferroni correction).

#### ROI-Based MVPA vs. Searchlight MVPA

Both ROI-based and searchlight MVPA are implemented in MVPANI. ROI-based MVPA refers to the MVPA procedure that is performed using all features in a specified ROI (defined using a mask file) and only one classification accuracy or prediction precision is obtained for the whole ROI. In contrast, searchlight MVPA is a “voxel-wise” MVPA procedure in which MVPA is performed for a small sphere centered at one voxel each time using this given voxel and all its neighboring voxels (i.e., a searchlight sphere) and then repeated for every voxel included in a predefined mask (Kriegeskorte et al., 2006). In this way, every voxel is assigned a classification accuracy or prediction precision, resulting in a brain map of classification accuracies or prediction precisions. The radius of the searchlight sphere may have effects on the amount of information contained in a searchlight sphere and also on the actual spatial resolution of the resultant classification accuracy map. A searchlight sphere with a larger radius contains more voxels and, thus, is more likely to contain more information that can be utilized in MVPA; meanwhile, the spatial resolution of the resultant accuracy map is lower because a bigger sphere covers a larger area, and thus, the information cannot be precisely localized in space. The radius of the searchlight sphere can be specified by users in MVPANI.

### “Output” Module

For classification, MVPANI automatically outputs the basic MVPA results, including the average classification accuracy, specificity, and sensitivity for classifications across all folds defined as follows:

$$\text{Accuracy}=\frac{T\,P+T\,N}{T\,P+F\,N+T\,N+F\,P}$$

$$\text{Sensitivity}=\frac{T\,P}{T\,P+F\,N}$$

$$\text{Specificity}=\frac{T\,N}{T\,N+F\,P}$$

where *TP* is the number of true positives (e.g., the number of patients correctly classified as patients), *TN* is the number of true negatives (e.g., the number of normal controls correctly classified as non-patients), *FP* is the number of false positives (e.g., the number of normal controls classified as patients), and *FN* is the number of false negatives (e.g., the number of patients classified as non-patients). For regression, the correlation coefficient of the predicted scores and the true scores (referred to as prediction precision here) is calculated as an output for a measure of performance (i.e., prediction precision). MVPANI also provides options to output the MVPA results for each fold, the average weight map across all folds, the weight map of each fold, the receiver operating characteristic (ROC) curve, and the area under ROC curve (AUC).

### “Statistical Testing” Module

Statistical testing is needed to test whether the obtained MVPA result (i.e., classification accuracy or prediction precision) is significantly higher than chance level. In MVPANI, the statistical significance of MVPA results is evaluated using a non-parametric permutation test (Golland and Fischl, 2003). For classification, a null distribution corresponding to classification accuracies obtained at chance level can be generated using a permutation test in the following steps: (1) the original labels of all training samples are randomly shuffled so that the newly assigned labels no longer contain the true class information of the training samples; (2) a classifier is trained using the same training samples but with random class labels; (3) the above trained classifier is used to predict the classes of test samples, and thus, a chance-level classification accuracy can be obtained; (4) this procedure is repeated *N* times to obtain a null distribution based on the *N* chance-level classification accuracies; (5) the true classification accuracy obtained using the true labels is compared with this null distribution to calculate the percentage of the chance-level accuracies that are equal to or greater than the true classification accuracy as the *P* value. For regression, a similar procedure is adopted with only one difference: The scores of all training samples are randomly permutated to generate a null distribution of predication precision, and consequently, the *P* value is calculated as the percentage of chance-level predication precisions that are equal to or greater than the true prediction precision. Note that, if none out of *N* permutations reached the actual classification accuracy (or prediction precision) obtained from the true labels (or scores), the *P* value should be denoted as *P* < 1/*N* instead of P = 0 because the precision of the calculated *P* values is dependent on the number of permutations. Usually, thousands or even more than 10 thousands of permutations are recommended for obtaining a more accurate *P* value, which might take a very long time to compute and sometimes becomes infeasible. Therefore, the number of permutations and the computation time need to be balanced in practice.

Importantly, we also provided an option for multiple comparison correction of the *P* values for searchlight MVPA. Indeed, for searchlight MVPA, classification or regression analysis is performed for each searchlight sphere, and thus, a large number of statistical tests need to be performed. To correct for this multiple testing problem, we use the following procedure based on permutation: (1) for each permutation step, MVPA is performed for every searchlight sphere using the same set of randomly assigned labels (or scores); (2) only the maximal classification accuracy (or prediction precision) across all searchlight spheres is kept for each permutation step; (3) after repeating this procedure for *N* times, *N* maximal classification accuracies (or prediction precisions) are obtained to build the null distribution for all searchlight spheres; (4) the true classification accuracy (or prediction precision) of every searchlight sphere is compared with this common null distribution to calculate the corrected *P* values for each searchlight sphere. This correction procedure corresponds to family-wise-error (FWE) correction.

### “Data Fusion” Module

**FIGURE 3:**
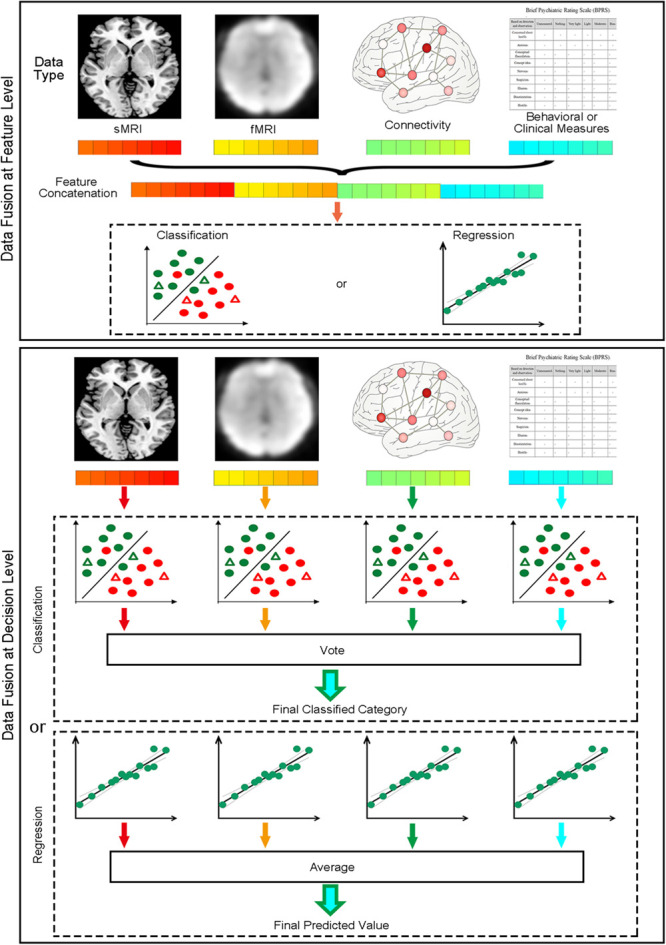
Two data fusion strategies implemented in MVPANI. The upper panel shows the feature concatenation strategy, where data are fused at the feature level by concatenating all different types of features into a single feature vector before feeding them into a classification or regression model. The lower panel shows the voting strategy, where data are fused at the decision level by building a classification or regression model for each type of data and then all models vote for the final decision (for classification, taking the majority of all models’ decisions; for regression, averaging the predicated values of all models).

When different types of data are available, MVPA can fuse different types of data containing complementary information to improve classification or regression performance. MVPANI offers two strategies for data fusion as shown in Figure 3. The first strategy is to fuse data at the feature level before MVPA by first building a fused feature vector by concatenating the feature vectors obtained from different data types and, second, feeding the fused feature vector to machine learning algorithms for classification or regression. Therefore, the final result is obtained using integrated data of different types. The functions of Load features and Concatenate features in the Data Fusion module allow users to enter all features and generate a single concatenated feature vector (saved as a .mat file in the current directory) for subsequent MVPA. Note that, as different types of data often have very different ranges, it is recommended that the data of each type should be normalized using, for example, *Z*-transformation, to a similar range before concatenating them into a single feature vector. In addition, as the fused feature vector is usually very large, dimension reduction or feature selection is recommended either before or after concatenation to reduce the feature dimension and computational load. We refer this strategy as Concatenate in MVPANI. The second strategy is to fuse data at the decision level. In this strategy, individual classification or regression models are built for each type of data separately, resulting in multiple classifiers or regression models. Each classifier or regression model makes its own decision for each test sample. Then all classifiers or regression models “vote” for the final decision using the Vote function in the Data Fusion module. That is, for classification, the final decision is determined by the majority of the decisions of all individual classifiers, and for regression, the final predicted value is determined by averaging the predicted values across all individual models. When voting or averaging, the weights of the decisions obtained from different types of data (usually the absolute decision value or the decision probability generated from the machine learning algorithms) are considered to assign more weight on the decisions that are made with more confidence.

For users’ convenience, we also provide the Load and Save function in MVPANI. The Save function allows users to save all specified configurations used in a particular MVPA task as a MATLAB data file (i.e., .mat format) for future use. The Load function allows users to load an existing configuration file (i.e., a .mat file) without having to go through every configuration step to configure their own MVPA analysis. This Save/Load function is particularly useful if users want to keep a record of what exact configurations they specified for a particular MVPA task or if they want to perform similar MVPA analysis many times (e.g., for different datasets each time or changing just one or two parameters each time).

## Applications of Mvpani

In this section, we use four examples to describe the usage of MVPANI in four different situations based on the following dataset.

**Dataset information:** The data are a part of the data used in a study that was approved by the Ethics Committee of Tianjin Medical University General Hospital. Twenty patients with subcortical infarcts (three females, 55 ± 9 years old) and 20 healthy controls (10 females, 59 ± 7 years old) participated in this study, and written informed consent was obtained from each participant before data collection. Note that we used a small sample size just for the purpose of demonstration. A large sample size usually gives more reliable results and is encouraged in real practice. Structural (T1 weighted, TR = 7.8 ms, voxel size = 1 × 1 × 1 mm) and resting-state functional MRI (rs-fMRI, TR = 2 s, voxel size = 3 × 3 × 3 mm, duration = 6 min) data were collected from all participants. The rs-fMRI data of each participant were preprocessed using the software package SPM8^2^ in the following steps: removing the first 10 volumes, slice timing, image realignment for head motion correction, regressing out nuisance covariates (six head motion parameters, the global signal, the white matter signal, and the cerebrospinal fluid signal), band-pass filtering (0.01–0.08 Hz), and spatial normalization to standard MNI space. Then three functional imaging metrics, including regional homogeneity (ReHo), fractional amplitude of low-frequency fluctuations (fALFF), and whole-brain functional connectivity (FC), were calculated from the preprocessed fMRI data for each participant using the software package REST (Song et al., 2011). After MRI scanning, all participants also performed a Flanker task during which participants were required to respond to the direction of a central arrow (target) and to ignore adjacent congruent or incongruent distracting arrows by pressing a button on the computer with their non-paretic hand (all patients suffered from hemiparesis at the acute stage of stroke). A total of 60 trials were presented, and response accuracy and reaction time (RT) of correct responses to the target were recorded. The T1 structural MRI data of each participant were analyzed using the software package VBM8^3^ to calculate the voxel-wise gray matter volume (GMV) and white matter volume (WMV). More detailed information can be found in our previous papers (Zhang et al., 2014; Diao et al., 2017; Liu et al., 2018).

### Example 1: Classification Between Patients and Controls

In this example, we used SVC to classify patients from controls based on whole-brain ReHo maps.

#### Input

Forty image files (.nii) corresponding to the ReHo maps of all participants were entered as input data. According to the alphabetic order of the file names of these image files, the first 20 files corresponded to patients, and the second 20 files corresponded to controls. We then created a label file (.xlsx) containing a column with “1” in the first 20 entries and “−1” in the second 20 entries (i.e., label “1” corresponding to patients and label “−1” corresponding to controls). To perform a leave-one-pair-out cross-validation, all participants were divided into 20 folds with one fold containing one patient and one control. The corresponding fold file (.xlsx) contained a column with 1, 2, 3, up to 20 in the first 20 entries and exactly the same numbers in the second 20 entries (i.e., the first patient and the first control were paired in the first fold). A whole-brain mask (a binary image containing only zeros and ones) was also specified using an image file (.nii).

#### Model Configuration

The ReHo images were used for the subsequent MVPA without any further feature preprocessing. Here, we used all voxels included in the whole-brain mask without feature selection. For the classification algorithm, we selected C-SVC with default parameter settings (linear kernel; penalty coefficient *c* = 1).

#### Output

**FIGURE 4:**
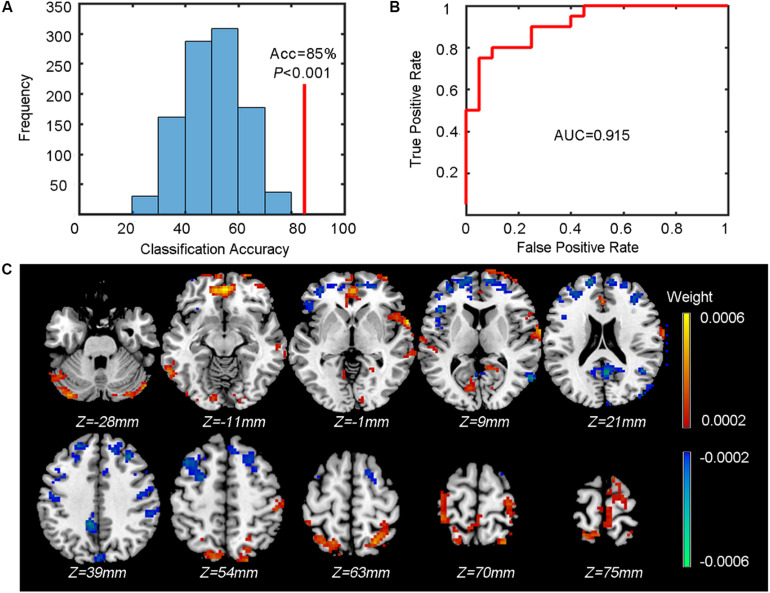
Results of the classification between patients and controls. **(A)** The classification accuracy (Acc, indicated by the red vertical line) and the *P* value obtained from the corresponding null distribution generated by 1000 permutations (indicated by the blue bell shape centered around chance level accuracy of 50%). **(B)** The ROC curve and the corresponding AUC. **(C)** The weight map (only voxels with the absolute value of weight over 0.0002 are shown). The color of each voxel indicates the weight (cold color indicates negative weights and warm color indicates positive weights).

The outputs include the classification accuracy (also with specificity and sensitivity), the ROC curve, and the mean weight map. We achieved a classification accuracy of 85% (indicated by the red vertical line in Figure 4A with a specificity of 75% and sensitivity of 95%. The corresponding ROC curve is shown in Figure 4B. Figure 4C shows the mean weight map obtained using the whole-brain mask (only the voxels with the absolute value of weight greater than 0.0002 were shown in the figure).

#### Statistical Testing

To test whether the obtained classification accuracy 85% was significantly higher than chance level, we also performed a permutation test (*n* = 1,000) to build up a null distribution of random classification accuracies. The comparison between the actual classification accuracy obtained from the true labels and the null distribution is shown in Figure 4A. We can see that none of the 1,000 permutations generated a classification accuracy equal to or greater than the actual accuracy, resulting in a *P* < 0.001 (i.e., *P* < 1/1000), indicating that the spatial patterns of ReHo images were distinguishable between patients and controls.

### Example 2: Information Mapping Using Searchlight MVPA

To localize the brain areas that contain sufficient information (i.e., distinct spatial patterns of activity) that can distinguish between patients and healthy controls based on ReHo maps, we performed a searchlight MVPA using SVC. The data used in this analysis is the same as the data used in Example 1.

#### Input

The image files (i.e., ReHo maps), the label and fold files, and the whole-brain mask file were the same as used in Example 1.

#### Model Configuration

The Searchlight option was checked to perform searchlight MVPA, and the size of the searchlight was defined as a sphere with a 4-voxel radius. The C-SVC with default parameter settings was used as the classification algorithm. No feature transformation or feature selection was applied.

#### Output

The main output was the classification accuracy map averaged across all cross-validation steps; that is, the value of each voxel indicates the average classification accuracy obtained using all voxels included in the predefined searchlight sphere centered at this given voxel.

#### Statistical Testing

**FIGURE 5:**
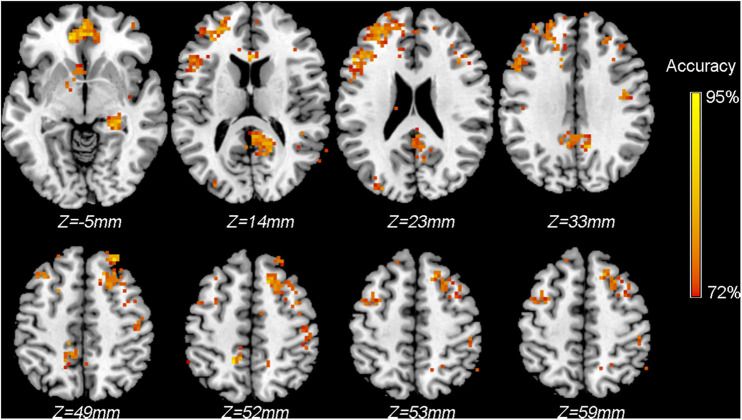
Classification accuracy map of the searchlight MVPA discriminating between patients and controls using ReHo maps. Colored voxels indicate centers of searchlight spheres where significantly higher-than-chance-level accuracies were obtained under the threshold of *P* < 0.005 (uncorrected, cluster >30 voxels). Values of colored voxels indicate the classification accuracies.

To identify the voxels with significantly higher than chance level classification accuracies, we performed a permutation test (*n* = 500). Figure 5 shows the voxels surviving the threshold of *P* < 0.005 (uncorrected, cluster >30 voxels). Note that, just for the purpose of demonstration, we only performed 500 permutations, and the result was not corrected for multiple comparisons as no voxels survived the correction. However, in real practice, a larger number of permutations (*n* ≥ 5000 is recommended) should be performed to ensure a reliable *P* value estimation; furthermore, uncorrected *P* values are not recommended.

### Example 3: Prediction of Reaction Time Using Brain Imaging Data

In this example, we demonstrate how to predict continuous values based on brain imaging data using regression in MVPANI. More specifically, we used fALFF maps obtained from rs-fMRI data to predict RTs to test whether resting-state brain activity can predict task performance.

#### Input

Only healthy subjects were included in this analysis. The RTs were averaged across all trials to indicate the task performance of a given participant. Therefore, 20 fALFF maps of healthy participants were entered as input data. We then created a label file containing the averaged RTs obtained from the 20 participants as a column. The order of participants in the label file corresponded to the participant order of the entered fALFF maps. To perform a leave-one-participant-out cross-validation, we also created a fold file containing a column with 1, 2, 3 up to 20 with each entry indicating a participant. A binary whole-brain gray matter mask was also specified.

#### Model Configuration

The fALFF images were used for the subsequent prediction analysis after data normalization for each sample using *Z*-transformation; that is, each sample (i.e., the data in each row of the data matrix) was normalized to have a mean of zero and a variance of one after normalization. We tested how feature selection could affect the prediction precision by selecting only the top voxels with highest *F* scores; the percentage of selected voxels ranged from 10% to 100% with a step of 20%. For the regression algorithm, we selected e-SVR with default parameter settings (linear kernel; penalty coefficient *c* = 1; the epsilon in loss function *p* = 0.1).

#### Output

**FIGURE 6:**
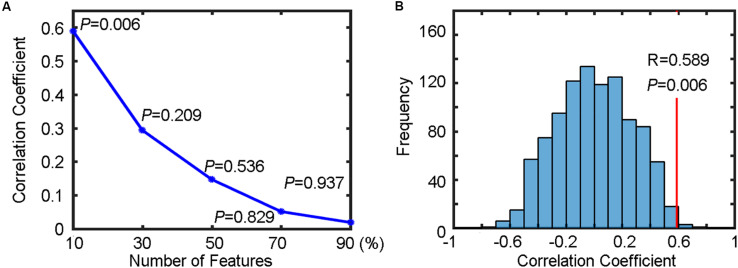
Regression results for predicting reaction times (RTs). **(A)** The change of correlation coefficients (along with their corresponding *P* values) with the number of selected features. **(B)** The correlation coefficient (*R* = 0.589, *P* = 0.006) between the predicted RTs and the actual RTs of a Flanker task (indicated by the red vertical line) and the corresponding null distribution generated by 1000 permutations (indicated by the blue bell shape centered around chance level correlation coefficient of 0).

The output results for each percentage of selected voxels included the predicted RTs and the correlation coefficient between the predicted RTs and the actual RTs. The change of correlation coefficients as a function of the percentage of selected voxels is shown in Figure 6A. We found that the prediction precision decreased drastically when the percentage of selected voxels increased and the highest prediction precision (*R* = 0.589) was obtained when only the top 10% of voxels were selected.

#### Statistical Testing

To test whether the obtained highest prediction precision (*R* = 0.589) was significantly higher than chance level, we performed a permutation test (*n* = 1000) to build a null distribution of correlation coefficients. The comparison between the actual correlation coefficient obtained from the true RTs and the corresponding null distribution is shown in Figure 6B. We found that six of 1000 permutations generated prediction precisions equal to or greater than the actual correlation coefficient (0.589), resulting in a *P* = 0.006. Because five different percentages for feature selection were tested (resulting in five independent SVR analyses), the above *P* value should be corrected for multiple comparisons to determine its statistical significant. Using Bonferroni correction, that is, *P* = 0.006 (< 0.05/5), the result can be considered as significantly higher than chance level, indicating that the spatial patterns of resting-state fALFF maps were predictive of the RTs of the flanker task in healthy participants.

### Example 4: Using Data Fusion to Improve Classification Performance

In this example, we demonstrate how to combine different measures extracted from multimodal neuroimaging data to improve performance of classification between patients and normal controls. We tested two data fusion strategies implemented in MVPANI; data fusion at the feature level using the feature concatenation strategy and data fusion at the decision level using the voting strategy and showed that the two data fusion strategies improved the classification performance in two examples.

#### Feature Concatenation Strategy

In this example, we combined fALFF maps (.nii files) and FC matrices (.mat files) from 20 patients and 20 controls. The two types of features, obtained from rs-fMRI data, represent different functional measures with different file formats. Using the Load and Concatenate functions in the Data Fusion module, a concatenated feature vector was generated and stored as a new file for each participant. These generated files were then entered as the input data files in the Input module. The label file, the fold file, and the mask file as used in Example 1 were also entered. For model configuration, C-SVC with default parameter settings as used in Example 1 were selected as the classification algorithm, and no feature transformation and feature selection was performed. Note that, here we did not perform data normalization before concatenation because the values of both fALFF and FC are within the range of -1 and 1. The classification accuracy obtained using the concatenated features was 82.25%, which was higher than the accuracies obtained from each type of features alone (80% accuracy when using fALFF maps and 70% accuracy when using FC matrices).

#### Voting Strategy

In this example, we combined ReHo maps, GMV maps, and WMV maps (the first was obtained from rs-fMRI data and the last two were obtained from structural data) to classify patients from healthy controls. Model configuration was identical to what was used in the example of the Feature concatenation strategy. We obtained a classification accuracy of 92.5% using the voting strategy, which was higher than the accuracies obtained from each type of features alone (85% accuracy when using ReHo maps, 87.5% accuracy when using GMV maps, and 90.0% accuracy when using WMV maps).

## Discussion

Here, we introduced a software package, named MVPANI, specifically designed for performing MVPA of neuroimaging data. MVPANI is featured with a number of advantages that make it a useful tool for neuroscientists and clinicians to take advantage of machine learning techniques in their research.

First, MVPANI has a GUI and, thus, is more friendly for researchers who are not experienced programmers. As previously noted, having a GUI also helps standardize the MVPA pipeline for neuroimaging data analysis and consequently makes it easier to replicate previous studies and compare results between different studies. MVPANI also offers the Save and Load functions in the GUI: With the Save function, researchers can easily save all configurations of their analysis pipeline so that they can log the exact settings for their analysis pipeline for future reference; with the load function, researchers can easily make modifications based on their previously used analysis pipelines and parameters and, thus, make it very convenient to compare results when changing a given parameter while maintaining all other parameters unchanged.

Second, MVPANI offers a Data fusion function so that researchers can combine different types of data when performing MVPA. Different types of data are often available in one study: (1) Multi-modal imaging data are usually collected in one study (e.g., structural T1 images and functional images); (2) even when only one imaging modality is used, different measures can be extracted (e.g., GMV, WMV, and cortical thickness can be extracted from the structural T1 images; ReHo, fALFF, FC, and effective connectivity can be extracted from the functional images; fractional anisotropy (FA), mean diffusivity (MD), and anatomical brain network can be extracted from diffusion data); (3) besides neuroimaging data, cognitive, behavioral, or even genetic data are often collected in neuroimaging studies as well. It is becoming a trend to integrate different types of data in neuroimaging studies as the information contained in a single type of data is always limited. Also, the two Data fusion methods (Feature Concatenation strategy and Vote strategy) currently implemented in MVPANI are also simple and easily understood and accepted in neuroimaging society. Therefore, the function of Data Fusion is very useful for researchers to utilize complementary information contained in different types of data to maximally exploit all available data in their research. Moreover, allowing the most universal file formats (.txt, .xlsx, and .mat) as input data formats makes MVPANI not limited in neuroimaging studies but is also suitable for applying machine learning techniques in other non-imaging (such as genetic) studies as well.

Third, MVPANI contains a variety of commonly used machine learning algorithms, currently including support vector machines, linear discriminant analysis, logistic regression, k-nearest neighbors, naive Bayes classifier, decision tree, and random forest, so that researchers can test different algorithms and identify the most suitable one for their own data. Furthermore, key parameters of each algorithm can be easily modified through GUI by users if needed.

Fourth, in addition to feature selection with a binary mask, MVPANI also offers several feature preprocessing steps including normalization, dimension reduction, and feature selection based on their discriminating ability, which have often been shown to be beneficial to MVPA performance.

Fifth, MVPANI is developed using MATLAB platform, which is already familiar to most neuroimaging researchers as many other popular software packages for neuroimaging data analyses, such as SPM, GIFT (Group ICA of fMRI Toolbox), DPABI, and so on, are also based on MATLAB platform. Importantly, sharing the same platform also makes it convenient to share codes between these software packages and perform integrated analyses using multiple software packages.

Sixth, MVPA results can be obtained in a variety of output formats, including classification accuracies, specificity, and sensitivity (for classification analysis) or prediction precisions (for regression analysis), the actual classification outcome or predicted values of each sample, the ROC curves with AUC, and the weight maps. For most of these result formats, MVPANI can output the overall results averaged across all cross-validation steps as well as the results of each cross-validation step so that researchers with limited programming skills can also examine the MVPA results in much more details just using the GUI.

Seventh, MVPANI offers the function of Statistical Testing of MVPA results. Without a convenient tool for statistical testing of MVPA results, previous studies often use *T*-tests to assess the statistical significance of the obtained classification accuracies or do not test statistical significance at all especially for searchlight analysis. As classification accuracies are not Gaussian distributed, traditional parametric statistical testing (e.g., one-sample *T*-test) is inappropriate in determining the statistical significance of MVPA results. The most commonly accepted statistical testing method is a non-parametric permutation test. Unlike *T*-tests, which has a known null distribution, a permutation test has to estimate the null distribution based on the real data with randomly shuffled labels of training samples and repeat the classification analysis many times with exactly the same model configurations. Therefore, common statistical software, such as SPSS cannot be used in this situation. Another issue with performing a permutation test is that it requires a large number of repetitions to build a reliable null distribution, which is computationally highly costly and time consuming. To make a large number of permutations feasible, parallel computing is adopted when running permutation test in MVPANI. Importantly, statistical significance can also be corrected for multiple comparisons to make the statistical testing more valid when multiple MVPA analyses are performed, e.g., in searchlight MVPA analysis. The permutation test with multiple comparisons correction implemented in MVPANI help make statistical testing of MVPA results a standard procedure in neuroimaging studies using machine learning.

Last, MVPANI is a free and open-source software package. It utilizes other existing software packages, and importantly, it is also readily to be utilized by other software developers. More advanced users can also use the functions implemented in MVPANI to create their own analysis pipelines to best fit their own research purpose.

Having mentioned all these advantages, it should be noted that the current version of MVPANI also has limitations and will be updated during its ongoing development. For example, only two-way classifications are implemented in the current version, and multi-class classifications will be implemented in the next version. Also, more complicated methods for data fusion with higher efficiency of information fusion from different types of data will be tested and implemented in MVPANI in the future. In addition, considering the facts that MVPA of neuroimaging data is computationally very expensive in general and GPU computing is becoming popular, we will also exploit GPU to boost the computation speed in the future, which is particularly useful for performing searchlight analysis with a large number of permutations. Furthermore, with its rapid development, deep learning is extracting more and more attention in neuroimaging field. We will also consider implementing some deep learning framework in future versions of MVPANI.

## Conclusion

MVPANI is a free and open source software toolkit for MVPA of neuroimaging-related data with a user-friendly GUI. It is easy to use and has a number of advantages compared with other existing software packages. Therefore, the development of MVPANI will encourage neuroimaging researchers to adopt machine learning techniques to complement traditional univariate analyses and fully exploit their data.

## Information Sharing Statement

The MVPANI software code is open source and can be downloaded from http://funi.tmu.edu.cn. The data for the examples can be accessed at http://funi.tmu.edu.cn. The MVPANI software comes with third party software (libsvm https://www.csie.ntu.edu.tw/~cjlin/libsvm/; TDT http://www.bccn-berlin.de/tdt; SPM http://www.fil.ion.ucl.ac.uk/spm/).

## Data Availability Statement

Publicly available datasets were analyzed in this study. This data can be found here: http://funi.tmu.edu.cn.

## Ethics Statement

This study was approved by the Ethics Committee of Tianjin Medical University General Hospital and informed consent was obtained from each participant before the study. All participants in this study provided written informed consent.

## Author Contributions

YP and ML contributed to the software design and manuscript writing. YP and XZ contributed to the software programming. YL, QS, and SW contributed to the software testing. FL contributed to the manuscript review and editing. ML and CY contributed to the supervision and funding provision. All authors approved the final version of the manuscript to be published.

## Conflict of Interest

The authors declare that the research was conducted in the absence of any commercial or financial relationships that could be construed as a potential conflict of interest.

## Footnotes

1 https://www.fil.ion.ucl.ac.uk/spm/

2 https://www.fil.ion.ucl.ac.uk/spm/

3 http://dbm.neuro.uni-jena.de/vbm8/
